# Targeting colon cancer stem cells using a new curcumin analogue, GO-Y030

**DOI:** 10.1038/bjc.2011.200

**Published:** 2011-06-21

**Authors:** L Lin, Y Liu, H Li, P-K Li, J Fuchs, H Shibata, Y Iwabuchi, J Lin

**Affiliations:** 1 Center for Childhood Cancer, The Research Institute at Nationwide Children's Hospital, Department of Pediatrics, Internal Medicine, College of Medicine, The Ohio State University, Columbus, OH 43205, USA; 2 Division of Cardiology, Department of Internal Medicine, Tongji Hospital, Tongji Medical College, Huazhong University of Science and Technology, Wuhan 430030, China; 3 Division of Medicinal Chemistry and Pharmacognosy, College of Pharmacy, The Ohio State University, Columbus, OH 43205, USA; 4 Departmentof Clinical Oncology, Graduate school of medicine, Akita University, Akita, Japan; 5 Department of Organic Chemistry, Graduate School of Pharmaceutical Sciences, Tohoku University, Aobayama, Sendai 980-8578, Japan

**Keywords:** STAT3, curcumin analogue, colon cancer, cancer stem cells, ALDH, CD133

## Abstract

**Background:**

Persistent activation of signal transducers and activators of transcription 3 (STAT3) is commonly detected in many types of cancer, including colon cancer. To date, whether STAT3 is activated and the effects of STAT3 inhibition by a newly developed curcumin analogue, GO-Y030, in colon cancer stem cells are still unknown.

**Methods:**

Flow cytometry was used to isolate colon cancer stem cells, which are characterised by both aldehyde dehydrogenase (ALDH)-positive and CD133-positive subpopulations (ALDH^+^/CD133^+^). The levels of STAT3 phosphorylation and the effects of STAT3 inhibition by a newly developed curcumin analogue, GO-Y030, that targets STAT3 in colon cancer stem cells were examined.

**Results:**

Our results observed that ALDH^+^/CD133^+^ colon cancer cells expressed higher levels of phosphorylated STAT3 than ALDH-negative/CD133-negative colon cancer cells, suggesting that STAT3 is activated in colon cancer stem cells. GO-Y030 and curcumin inhibited STAT3 phosphorylation, cell viability, tumoursphere formation in colon cancer stem cells. GO-Y030 also reduced STAT3 downstream target gene expression and induced apoptosis in colon cancer stem cells. Furthermore, GO-Y030 suppressed tumour growth of cancer stem cells from both SW480 and HCT-116 colon cancer cell lines in the mouse model.

**Conclusion:**

Our results indicate that STAT3 is a novel therapeutic target in colon cancer stem cells, and inhibition of activated STAT3 in cancer stem cells by GO-Y030 may offer an effective treatment for colorectal cancer.

Colorectal cancer is the third leading cause of cancer-related deaths in the United States. For patients with advanced colon cancer, the 5-year survival rate is less than 10%. Recent evidence suggests the existence of a small population of tumourigenic stem cells responsible for tumour initiation, metastasis and resistance to chemotherapy and radiation. Increasing evidence suggests that cancer stem cells are also relevant to colorectal cancer, and that they have an important role in cancer spread and recurrence (Barker *et al*, 2007; O'Brien *et al*, 2007; Ricci-Vitiani *et al*, 2007; Boman and Huang, 2008). It is important to identify the regulatory mechanisms and signalling pathways involved in colon cancer stem cells and develop novel reagents to target this refractory colon cancer stem cell population.

The signal transducers and activators of transcription (STAT) protein family represents a group of transcription factors that have a role in relaying extracellular signals initiated by cytokines and growth factors from the cytoplasm to the nucleus (Calo *et al*, 2003; Frank, 2007; Germain and Frank, 2007). Following activation, phosphorylated STATs dimerise and translocate to the nucleus, where they regulate the expression of numerous critical genes involved in cell cycle progression, proliferation, invasion, and survival. However, the constitutive activation of STAT3 is frequently detected in primary human cancer cells, including colorectal carcinoma cells (Corvinus *et al*, 2005; Kusaba *et al*, 2005). Persistent STAT3 activation is associated with enhanced proliferation and invasion of colorectal cancer cells *in vitro* and tumour growth in a colorectal tumour model *in vivo*, and inhibition of STAT3 induces apoptosis and reduces tumour cell invasion in colorectal cancer cells (Corvinus *et al*, 2005; Lin *et al*, 2005; Tsareva *et al*, 2007; Xiong *et al*, 2008). These reports indicate that constitutively activation of STAT3 is one of the important pathways that contribute to the oncogenesis in colorectal cancer and can serve as an attractive therapeutic target for colorectal carcinoma.

During the past decade, a number of developmental pathways that regulate cancer stem cells, especially in breast cancer stem cells, have been elucidated. These pathways include Notch, Hedgehog, Wnt, human epidermal growth factor receptor 2, AKT etc (Liu and Wicha, 2010). However, the role of STAT3 in colon cancer stem cells and the effect of STAT3 inhibition in colon cancer stem cells are still unknown.

Many markers and features of cancer stem cells have been defined. The transmembrane protein CD133 (Prominin-1 or AC133) is one of the markers that was first used to identify and isolate stem cells in brain cancers (Singh *et al*, 2004). Subsequently, CD133 was used to isolate stem cells from a host of other normal and cancerous tissues, including colon cancer (O'Brien *et al*, 2007; Ricci-Vitiani *et al*, 2007). Another potential colon cancer stem cell marker is aldehyde dehydrogenase 1 (ALDH1), a detoxifying enzyme that oxidises intracellular aldehydes and thereby confers resistance to alkylating agents (Magni *et al*, 1996; Yoshida *et al*, 1998). Implantation of as few as 100 ALDH^+^ cells was capable of tumour initiation (Huang *et al*, 2009). When using ALDH and CD133 together to form tumour xenografts, ALDH^+^/CD133^+^ cells showed an increased ability to generate tumour xenografts compared with ALDH^+^/CD133^–^ or ALDH^+^ alone (Huang *et al*, 2009). The present study uses both ALDH and CD133 together as markers for colorectal stem cells and examines the role of the STAT3 pathway in these cancer stem cells. Our results indicated that ALDH^+^/CD133^+^ subpopulation of colorectal cancer stem cells expressed higher levels of STAT3 phosphorylation compared with ALDH^−^/CD133^–^ subpopulations.

Curcumin is the primary bioactive compound isolated from turmeric, the popular Indian curry spice. Curcumin has anti-inflammatory, antioxidant, chemopreventive and chemotherapeutic properties by regulating multiple cell signalling pathways, including the STAT3 pathway (Aggarwal and Shishodia, 2006). It has been used against various types of cancers, including colon cancer, with little to no toxicity (Hatcher *et al*, 2008). Our results indicated that curcumin inhibited STAT3 phosphorylation, cell viability, and tumoursphere formation in ALDH^+^/CD133^+^ colon cancer stem cells. A novel curcumin analogue, GO-Y030 (Shibata *et al*, 2009), also inhibited STAT3 phosphorylation, the expression of STAT3 downstream target genes, cell viability, tumoursphere-forming capacity, and induced apoptosis in ALDH^+^/CD133^+^ cells. The effects of GO-Y030 were more potent than curcumin. Furthermore, GO-Y030 inhibited tumour growth of ALDH^+^/CD133^+^ cells in the mouse model *in vivo*. Our results suggest that STAT3 is a novel therapeutic target in colorectal cancer stem cells, and the novel curcumin analogue, GO-Y030, might be used as a new therapeutic reagent to target colon cancer stem cells in future.

## Materials and methods

### Colon cancer cell lines

Human colorectal cancer cell lines (DLD-1, HCT-116, SW480, and HT29) were purchased from the American Type Culture Collection (Manassas, VA, USA) and maintained in Dulbecco's Modified Eagle Medium supplemented with 10% fetal bovine serum (FBS), 4.5 g l^−1^ L-glutamine, sodium pyruvate, and 1% penicillin/streptomycin. All cell lines were stored in a humidified 37°C incubator with 5% CO_2_.

### GO-Y030 and curcumin

Curcumin was purchased from Sigma-Aldrich (St Louis, MO, USA). GO-Y030 (Supplementary Figure 1), a new curcumin analogue (Shibata *et al*, 2009), was provided by Dr Shibata's laboratory.

### Computational binding studies of GO-Y030

Molecular docking program MLSD based on AutoDock 4 was used to dock GO-Y030 to the binding sites of the STAT3 SH2 domain (PDB code 1BG1). ADT tool was used to prepare parameter and input files as previously reported (Huey *et al*, 2007; Li and Li, 2010). The small molecule GO-Y030 was docked to STAT3 SH2 using Lamarckian Genetic Algorithms (LGA) and Particle Swarm Optimisations (PSO) as searching methods. Docking simulations were repeated for 100 runs, with 1.0 and 6.5 million energy evaluations being used for PSO and LGA, respectively, in each run. The Docking found a few distinct conformational clusters. The binding modes of GO-Y030 were clustered with an RSMD of 2.0 Å. The major clusters with top binding energies were visually examined for binding modes.

### Cell viability assay

Colon cancer stem cells (3000 per well in 96-well plates) were incubated with desired concentrations of compounds in triplicate at 37°C for 72 h. 3-(4,5-Dimethylthiazolyl)-2,5-diphenyltetrazolium bromide viability assays were performed and the absorbance was read at 595 nm. Half-maximal inhibitory concentrations (IC_50_) were determined using Sigma Plot 9.0 Software (Systat Software Inc., San Jose, CA, USA).

### Isolation of cancer stem cells

The ALDEFLUOR kit (StemCell Technologies, Durham, NC, USA) was used to isolate subpopulations with high ALDH enzymatic activity as previously described (Ginestier *et al*, 2007). Briefly, cells were trypsinised to single cells using 0.05% trypsin and subsequently suspended in ALDEFLUOR assay buffer containing ALDH substrate (BAAA, 1 *μ*mol l^−1^ per 1 × 10^6^ cells) and then incubated for 40 min at 37°C. For each sample, an aliquot of cells was stained under identical conditions with 15 mmol l^−1^ diethylaminobenzaldehyde (DEAB), a specific ALDH inhibitor, as a negative control. In all experiments, the ALDEFLUOR-stained cells treated with DEAB served as ALDH-negative controls. Anti-human PE-CD133 antibody (Miltenyi Biotec, Auburn, CA, USA) were used to identify CD133-positive cells. ALDH^+^/CD133^+^ and ALDH^−^/CD133^−^ subpopulations were separated from SW480, HCT116, DLD-1, and HT29 colon cancer cells by a FACS Wantage SE (Becton Dickinson, Palo Alto, CA, USA) Flow Cytometer. After sorting, ALDH^+^/CD133^+^ cells were cultured in serum-free stem cell medium (mammary epithelial basal medium) to maintain cancer stem cell characteristics. Cancer stem cells were grown in a serum-free mammary epithelial basal medium (Clonetics division of Cambrex BioScience, Walkerville, MD, USA) supplemented with B27 (Invitrogen, Carlsbad, CA, USA), 20 ng ml^−1^ EGF (BD Biosciences, San Jose, CA, USA), antibiotic–antimycotic (100 U ml^−1^ penicillin-G sodium, 100 *μ*g ml^−1^ streptomycin sulphate), 4 *μ*g ml^−1^ gentamycin, 1 ng ml^−1^ hydrocortisone, 5 *μ*g ml^−1^ insulin, and 100 *μ*M *β*-mercaptoethanol (Sigma-Aldrich) in a humidified incubator (5% CO_2_) at 37°C. ALDH^−^/CD133^−^ cells and un-separated cells were cultured in regular medium and replaced with the stem cell medium above for 3 days before harvesting.

### Western blot analysis

After treatment with GO-Y030 (5 *μ*M or 10 *μ*M) or DMSO for 24 h, ALDH^+^/CD133^+^, ALDH^−^/CD133^−^ and un-separated DLD-1, HCT-116, SW480, and HT29 colorectal cancer cells were lysed in cold RIPA lysis buffer containing protease inhibitors and subjected to SDS–PAGE. Proteins were transferred to a PVDF membrane and probed with antibodies (Cell Signaling Tech., Danvers, MA, USA). Membranes were probed with a 1 : 1000 dilution of antibodies (Cell Signaling Tech.) against phospho-specific STAT3 (Tyrosine 705), phospho-independent STAT3, phospho-specific ERK1/2 (Threonine 202/Tyrosine 204, T202/Y204), cleaved caspase-3, cleaved PARP, Phospho-Rb (Ser780), and GAPDH. Membranes were analysed using enhanced chemiluminescence Plus reagents and scanned with the Storm Scanner (Amersham Pharmacia Biotech Inc., Piscataway, NJ, USA). The intensity of bands was quantified and normalised to GAPDH. For interferon-*γ* (IFN-*γ*), IL-4, and IL-6 stimulation experiments, HT29 colon cancer cells were serum-starved for 24 h and left untreated or pre-treated with GO-Y030 (2.5–10 *μ*M) or DMSO for 2 h. Then, 50 ng ml^−1^ IFN-*γ*, IL-4, or IL-6 were added and the cells were harvested for western blot analysis 30 min later.

### Reverse transcriptase–polymerase chain reaction

ALDH^+^/CD133^+^ subpopulations of DLD-1, HCT-116, and SW480 colon cancer cells were treated with GO-Y030 (5 *μ*M) or DMSO for 24 h. RNA was then collected using RNeasy Kits (Qiagen, Valencia, CA, USA). Primer sequences and source information of STAT3 downstream target genes can be found in Supplementary Table 1.

### Annexin-V apoptosis assay

Apoptotic cell death induced by GO-Y030 was quantified by flow cytometry with Annexin-V/propidium iodide (PI) double staining (BD Pharmingen, San Jose, CA, USA). After treatment with GO-Y030 or DMSO for 48 h, ALDH^+^/CD133^+^ SW480 colon cancer stem cells were collected and washed with cold PBS. The cell pellet was then re-suspended in 1 × binding buffer. Annexin V-FITC and PI (5 *μ*l per 100 *μ*l buffer) were added for 15 min at room temperature (RT) in darkness, and then analysed by flow cytometry (Becton Dickinson, Franklin Lakes, NJ, USA) within 1 h.

### Tumoursphere culture

The ALDH^+^/CD133^+^ and ALDH^−^/CD133^−^ subpopulations of DLD-1, HCT-116, SW480, and HT29 colorectal cancer cells were plated as single cells in ultra-low attachment six-well plates (Corning, Lowell, MA, USA) at a density of 250 to 50 000 viable cells per well in duplicate. Cells were grown in a serum-free stem cell medium described as above in a humidified incubator (5% CO_2_) at 37°C. On the second day after seeding, the ALDH^+^/CD133^+^ cells were treated with 2.5–5 *μ*M of GO-Y030. Tumourspheres were observed under microscope 10 to 15 days later. For counting tumourspheres, the content of all wells was collected, pooled, and transferred onto a collagen-coated six-well dish in differentiating medium (DMEM supplemented with 10% FBS). Tumourspheres adhered in these conditions in approximately 24 h, after which they were stained with crystal violet and counted under low magnification.

### Mouse xenograft tumour model

Animal studies were conducted in accordance with the principles and standard procedures approved by IACUC at the Research Institute at Nationwide Children's Hospital. SW480 and HCT-116 ALDH^+^/CD133^+^ cells (1 × 10^5^) were injected subcutaneously into the right flank area of 4- to 5-week-old female, non-obese diabetic/severe combined immunodeficiency (NOD/SCID) mice, which were purchased from Jackson Laboratory (Bar Harbor, ME, USA). After 10 days, mice were divided into two treatment groups consisting of six mice per group: Control vehicle (100% DMSO) and 50 mg kg^−1^ of GO-Y030. Tumour growth was determined by measuring the length (L) and width (W) of the tumour every other day with a caliper and tumour volume was calculated on the basis of the following formula: volume=(π/6) LW^2^.

## Results

### The phosphorylation of STAT3 in ALDH^+^/CD133^+^ subpopulation of colorectal cancer cells compared with the ALDH^–^/CD133^–^ subpopulation

**Figure 1:**
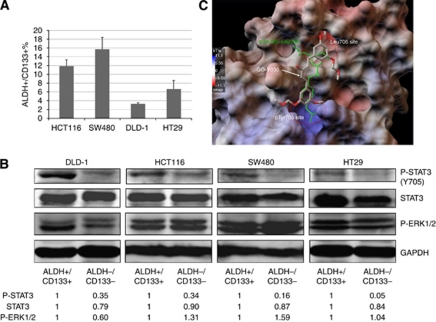
STAT3 phosphorylation of ALDH^+^/CD133^+^ subpopulation of colon cancer cells is higher than un-separated and the ALDH^−^/CD133^−^ subpopulations. (**A**) ALDH^+^/CD133^+^ and ALDH^−^/CD133^−^ subpopulations were separated from DLD-1, HCT-116, and SW480 colon cancer cells by flow cytometer. The percentage of ALDH^+^/CD133^+^ subpopulations was shown. (**B**) Phosphorylation of STAT3 (Y705), ERK 1/2 (T202/Y204), phospho-independent STAT3 of ALDH^+^/CD133^+^, and ALDH^−^/CD133^−^ subpopulations were detected by western blot. (**C**) Computer modelling of GO-Y030 binding to STAT3 SH2 domain. GO-Y030 is in Thick Stick-Ball (S-B) model and in grey colour. The native pTyr–Leu706 phospho-peptide binding of the partnering SH2 in homo-dimerisation is in green colour. GO-Y030 occupied both pTyr705- and Leu706-binding sites, which very effectively displaced the native pTyr705–Leu706 peptide with a stronger binding affinity than native peptide in the binding site of STAT3 SH2 domain. The colour reproduction of this figure is available at the *British Journal of Cancer* online.

To determine whether STAT3 is activated in colorectal cancer stem cells, we separated ALDH^+^/CD133^+^ and ALDH^−^/CD133^−^ subpopulations from DLD-1, HCT-116, SW480, and HT29 colorectal cancer cell lines by flow cytometry, as previously described (Ginestier *et al*, 2007). The percentage of ALDH^+^/CD133^+^ subpopulations from HCT-116, DLD-1, SW480, and HT-29 colon cancer cells were shown in Figure 1A. ALDH+/CD133+ subpopulations of colorectal cancer cells have been reported as having an increased ability to generate tumour xenografts compared with ALDH^+^/CD133^–^ or ALDH^+^ alone, and exhibits properties of colorectal cancer stem cells *in vitro* and *in vivo* (Huang *et al*, 2009). To confirm the cancer stem cell properties of ALDH^+^/CD133^+^ subpopulations, we first compared the tumourspere-forming ability between ALDH^+^/CD133^+^ and ALDH^−^/CD133^−^ subpopulations. As shown in Table 1 and Supplementary Figure 2, ALDH^+^/CD133^+^ cells of SW480, HCT-116, DLD-1, and HT29 all generated more tumoursperes than ALDH^−^/CD133^−^ cells. We also tested the expression of other stem cell markers, such as CD44, Oct-4, and Nestin in ALDH^+^/CD133^+^ and ALDH^−^/CD133^−^ cells (Supplementary Figure 3). CD44 expression of ALDH^+^/CD133^+^ was higher than ALDH^−^/CD133^−^cells. However, Oct-4 expression was lower and there was no difference in Nestin expression between them. There are a few papers that reported CD44 as well as ALDH and CD133 are markers of colon cancer stem cells (Dalerba *et al*, 2007; Du *et al*, 2008; Todaro *et al*, 2010). To date, the experimental data to support Oct-4 and Nestin as colon cancer stem cell markers are still lacking.

The level of STAT3 phosphorylation at Tyrosine residue 705 (Y705) was then examined in ALDH^+^/CD133^+^ and ALDH^−^/CD133^−^ cells. Interestingly, our results showed that the ALDH^+^/CD133^+^ subpopulations of SW480, HCT-116, DLD-1, and HT29 (Figure 1B) colorectal cancer cells expressed higher levels of STAT3 phosphorylation (Y705) compared with the ALDH^−^/CD133^−^ subpopulation cells. The phosphorylation of ERK (Threonine 202/Tyrosine 204) in the ALDH^+^/CD133^+^ subpopulations was not higher than that of ALDH^−^/CD133^−^ subpopulations in the all four cell lines. Here we demonstrated that colorectal cancer stem cells (ALDH^+^/CD133^+^cells) expressed higher phosphorylated or activated STAT3 compared with ALDH^−^/CD133^−^ cells. These results suggested that the STAT3 pathway has a more important role in colorectal cancer stem cells.

### Computational binding modelling of GO-Y030

GO-Y030 is a newly development curcumin analogue (Supplementary Figure 1; Shibata *et al*, 2009). It has been demonstrated to inhibit colorectal carcinoma cells growth *in vitro* and in a mouse model *in vivo* (Shibata *et al*, 2009). However, the mechanism of GO-Y030 inhibition of colorectal carcinogenesis is still not very clear. We previously reported that Curcumin analogue GO-Y030 inhibits STAT3 activity and cell growth in breast and pancreatic carcinomas (Cen *et al*, 2009). Here, we used molecular docking program MLSD based on the AutoDock 4 to investigate that if GO-Y030 binds to the STAT3 SH2 domain. In a major conformational cluster, GO-Y030 occupied both the pTyr705 and Leu706 binding sites in the STAT3 SH2 domian, which contributed a binding energy of −8.2 kcal mol^−1^ (Figure 1C). GO-Y030 binding to both pTyr705 and Leu706 binding sites could displace the native pTyr705–Leu706 peptide more effectively than the binding of Curcumin to pTyr705 and the side pocket (Figure 1C).

### GO-Y030 inhibited the STAT3 phosphorylation in colorectal cancer cells

To confirm the inhibition of phosphorylated or activated STAT3 by GO-Y030 in colon cancer cells, we examined STAT3 phosphorylation (Y705) in three independent colon cancer cell lines (cells were cultured in 10% FBS) using phospho-STAT3 (Tyrosine 705) antibodies (Supplementary Figure 4). Phosphorylation at Y705 is important in the activation of STAT3 (Kaptein *et al*, 1996; Schaefer *et al*, 1997; Faruqi *et al*, 2001). Our results indicated that GO-Y030 significantly inhibited STAT3 phosphorylation (Y705) in DLD-1, HCT-116, and SW480 human colon cancer cell lines (Supplementary Figure 4). The inhibition of STAT3 phosphorylation by GO-Y030 was consistent with the induction of apoptosis, as evidenced by the cleavages of PARP and caspase-3 (Supplementary Figure 4).

There are seven known mammalian STAT proteins (1–4, 5a, 5b, and 6), which can be activated by certain cytokines or growth factors (Turkson and Jove, 2002; Calo *et al*, 2003; Frank, 2007; Germain and Frank, 2007). After activation, STAT1 regulates the expression of genes that promote growth arrest and apoptosis, and is considered as a putative tumour suppressor (Calo *et al*, 2003; Yu *et al*, 2009). STAT3 and STAT6 are involved in inhibiting anti-tumour immunity (Yu *et al*, 2009). To investigate the specific inhibition of GO-Y030, we detected the phosphorylation of STAT3, STAT1, or STAT6 induced by IL-6, IFN-γ, or IL-4 in HT29 colon cancer cell lines. GO-Y030 inhibited un-induced (Supplementary Figure 4) and IL-6 (50 ng ml^−1^)-induced phosphorylation of STAT3 (Y705) (Supplementary Figure 5A). However, GO-Y030 did not inhibit phosphorylation of STAT1 or STAT6 induced by 50 ng ml^−1^ of IFN-*γ* or IL-4 (Supplementary Figures 5B, 5C). This indicated the selectivity of GO-Y030 on STAT3, but not STAT1 and STAT6. The inhibition of STAT3 phosphorylation by GO-Y030 is unlikely through JAK2, as JAK2 phosphorylation is not reduced (Supplementary Figure 5A).

### GO-Y030 inhibited STAT3 phosphorylation and induced apoptosis in ALDH^+^/CD133^+^ subpopulations of colorectal cancer cells

**Figure 2:**
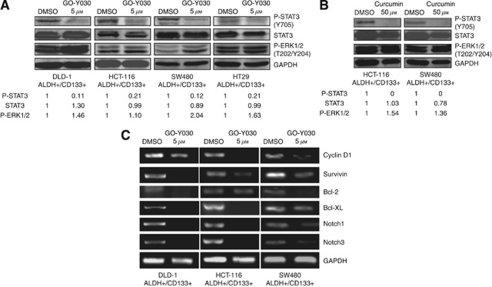
GO-Y030 inhibited STAT3 phosphorylation and downregulated STAT3-regulated genes expression in ALDH^+^/CD133^+^ cells. (**A**) ALDH^+^/CD133^+^ cells were treated with DMSO or 5 *μ*M of GO-Y030 for 24 h. Phosphorylation of STAT3 in DLD-1, HCT-116, and SW480 colon cancer stem cells were detected by western blot. (**B**) ALDH^+^/CD133^+^ cells were treated with DMSO or 50 *μ*M of curcumin for 24 h. (**C**) ALDH^+^/CD133^+^ cells were treated with GO-Y030 (5 *μ*M) or DMSO for 24 h. Reverse transcriptase–polymerase chain reaction reveals decreased expression of STAT3 downstream target genes in GO-Y030-treated cells as compared with DMSO control.

To confirm the important role of STAT3 in colon cancer stem cells, we next examined the effect of GO-Y030 in colorectal cancer stem cells. We observed that GO-Y030 inhibited STAT3 phosphorylation (Y705), but not ERK1/2 phosphorylation (T202/Y204) in the ALDH^+^/CD133^+^ subpopulation of SW480, HCT-116, DLD-1, and HT29 (Figure 2A) colorectal cancer cell lines. Curcumin also inhibited STAT3 phosphorylation (Y705) in the ALDH^+^/CD133^+^ subpopulations of SW480 and HCT-116 colorectal cancer cell lines (Figure 2B) at higher concentration (50 *μ*M). These results indicated that GO-Y030 was a potent inhibitor of STAT3 phosphorylation in these colorectal cancer stem cells. GO-Y030 also reduced the percentage of ALDH^+^/CD133^+^ cells in HCT-116 and SW480 colorectal cancer cell lines (Supplementary Figure 6).

The inhibition of STAT3 by GO-Y030 also downregulated the expression of many known STAT3-regulated genes in colorectal cancer stem cells related to cancer cell proliferation, survival, and angiogenesis, such as Cyclin D1 (Bromberg *et al*, 1999), survivin (Gritsko *et al*, 2006), Bcl-2, and Bcl-XL (Bromberg *et al*, 1999; Figure 2C). Furthermore, GO-Y030 inhibited Notch-1 and Notch-3 expression (Figure 2C) in ALDH^+^/CD133^+^ cells, which have recently been reported as a putative STAT3 downstream target gene (Grivennikov and Karin, 2008). The Notch signalling pathway is known to be essential for normal stem cell self-renewal and differentiation in a variety of tissues, and is involved in human cancer stem cells' self-renewal capacity and tumourigenicity (Dontu *et al*, 2004; Grivennikov and Karin, 2008).

**Figure 3:**
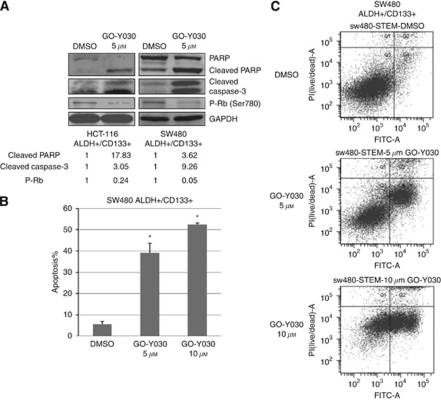
GO-Y030 induced apoptosis in ALDH^+^/CD133^+^ cells. (**A**) GO-Y030 increased the expression of cleaved PARP and cleaved caspase-3 and inhibited RB phosphorylation (Ser780) in HCT116 and SW480 colon cancer stem cells. (**B**, **C**) After treatment with GO-Y030 or DMSO for 48 h, ALDH^+^/CD133^+^ SW480 colon cancer stem cells were collected and analysed by flow cytometry. GO-Y030 led to a dose-dependent increase in apoptosis (^*^P<0.05).

We further detected the effect of GO-Y030 on colon cancer stem cell apoptosis and cell cycle. The results showed that GO-Y030 increased the expression of cleaved PARP and cleaved caspase-3, which indicated cell apoptosis (Figure 3A). GO-Y030 also inhibited RB phosphorylation (Ser780), which should arrest cell cycle progression in G1 in HCT116 and SW480 colon cancer stem cells (Figure 3A). The effects of GO-Y030 on colon cancer stem cell apoptosis was also detected by flow cytometry after staining with Annexin-V/PI. The results showed that GO-Y030 led to a dose-dependent increase in apoptosis. The percentage of apoptosis cells increased from 5.3±1.3 to 39.1±4.6% (5 *μ*M GO-Y030, *P*<0.05) and 52.4±0.8% (10 *μ*M GO-Y030, *P*<0.05) in SW480 colon cancer stem cells (Figures 3B and C). These results indicated that GO-Y030 induces apoptosis and cell cycle arrest in colon cancer stem cells.

### GO-Y030 inhibited cell viability and tumoursphere-forming capacity of ALDH^+^/CD133^+^ cells

**Figure 4:**
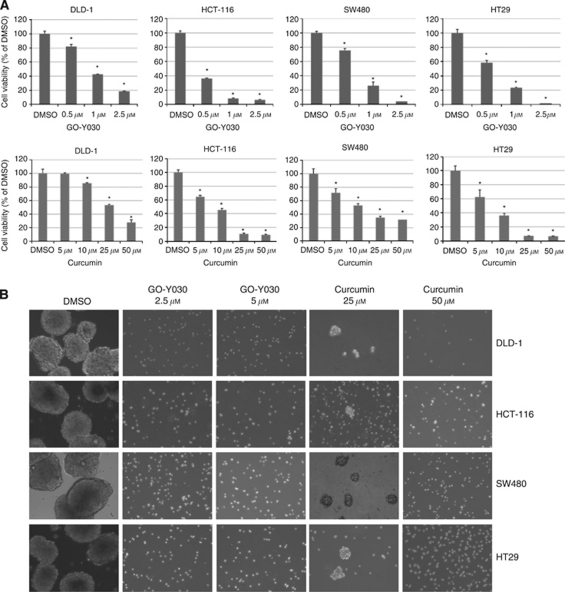
GO-Y030 and curcumin inhibited cell viability and tumoursphere formation of colon cancer stem cells. (**A**) The ALDH^+^/CD133^+^ cells were seeded in 96-well plates (3000 cells per well) in triplicates in a serum-free mammary epithelial basal medium (MEBM). The following day, cancer stem cells were treated with 0.5 to 2.5 *μ*M of GO-Y030 or 5–50 *μ*M of curcumin for 72 h. At the end of each time point, 3-(4,5-dimethylthiazolyl)-2,5-diphenyltetrazolium bromide assay was used to determine cell viability (^*^P<0.05). (**B**) The ALDH^+^/CD133^+^ cells were plated as single cells in ultra-low attachment six-well plates (Corning) at a density of 50 000 viable cells per well. Cells were grown in a serum-free MEBM as described in Materials and Methods. Twenty-four hours after seeding, the ALDH^+^/CD133^+^ cells were treated with 2.5 or 5 *μ*M of GO-Y030 or 25 or 50 *μ*M of curcumin.

We next examined the inhibitory effects of GO-Y030 and curcumin on cell viability in colorectal cancer stem cells. Our results observed that GO-Y030 and curcumin could inhibit cell viability of the ALDH+/CD133+ subpopulation from SW480, HCT-116, DLD-1, and HT29 (Figure 4A) colorectal cancer cells, further supporting the idea that this subpopulation of colorectal cancer stem cells is sensitive to GO-Y030. GO-Y030 was more potent than curcumin in inhibiting cell viability of the ALDH+/CD133+ subpopulations from SW480, HCT-116, DLD-1, and HT29 (Figure 4A). We compared the IC_50_ of colon cancer cells with tumour stem cells after GO-Y030 treatment in Supplementary Table 2. There is no significant difference between the IC_50_ values, they are both sensitive to GO-Y030. Furthermore, we examined the efficacy of GO-Y030 in inhibiting colorectal cancer stem cells to survive and proliferate in anchorage-independent conditions and their ability to form tumourspheres. Our results indicated that GO-Y030 and curcumin can inhibit tumoursphere-forming capacity in the ALDH+/CD133+ subpopulation of SW480, HCT-116, DLD-1, and HT29 (Figure 4B) colorectal cancer cells. Again, we also found that GO-Y030 was more potent than curcumin (Figure 4B). The GO-Y030-treated cells remaining in the plates were not viable as verified by Trypan blue exclusion assay (data not shown). Therefore, we demonstrated that colorectal cancer stem cells in the ALDH^+^/CD133^+^ cells expressed an activated form of STAT3, and this is the first report that demonstrates that these cancer stem cells are sensitive to GO-Y030 inhibition. These results indicated that GO-Y030 was a good drug candidate for targeting colorectal cancer stem cells for inhibition of phosphorylated or activated STAT3.

### GO-Y030 suppresses tumour growth of colon cancer stem cells in the mouse tumour model

**Figure 5:**
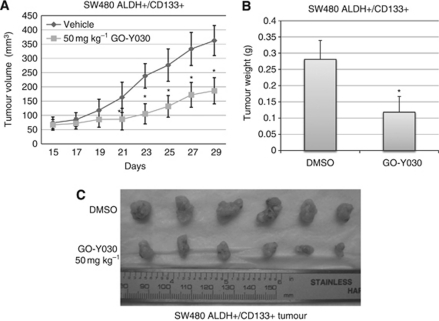
(**A**) GO-Y030 suppressed tumour growth in mouse xenografts with SW480 colon cancer stem cells. The mice were given daily intraperitoneal dosages of 50 mg kg^−1^ GO-Y030 or DMSO. Tumour volume (**A**), tumour weight (**B**), and tumour mass (**C**) were reduced in GO-Y030-treated mice compared with DMSO vehicle group (^*^P<0.05).

**Figure 6:**
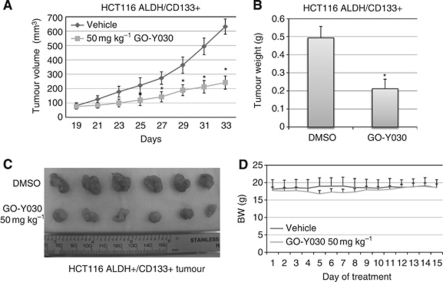
(**A**) GO-Y030 suppressed tumour growth in mouse xenografts with HCT-116 colon cancer stem cells. The mice were given daily intraperitoneal dosages of 50 mg kg^−1^ GO-Y030 or DMSO. Tumour volume (**A**), tumour weight (**B**), and tumour mass (**C**) were reduced in GO-Y030-treated mice compared with DMSO vehicle group (^*^P<0.05). (**D**) The reduction of bodyweights of GO-Y030-treated mice was similar to that of the vehicle-treated mice over 15 days of treatments.

We have demonstrated that GO-Y030 inhibits STAT3 phosphorylation, cell viability, and tumoursphere growth in colorectal cancer stem cells expressing elevated levels of STAT3 phosphorylation *in vitro*. To determine whether GO-Y030 may have therapeutic potential for clinical colorectal carcinoma treatment, we further tested GO-Y030 against ALDH^+^/CD133^+^ cells isolated from the SW480 and HCT-116 colon cancer cells in NOD/SCID mice xenograft models *in vivo*. SW480 and HCT-116 cancer stem cells (1 × 10^5^ cells per mouse) were injected subcutaneously into nude mice in two groups, DMSO vehicle group with six mice and GO-Y030 group with six mice. GO-Y030 (50 mg kg^−1^) was administrated via intraperitoneal injection beginning on day 15 or day 19. Caliper measurements of the longest perpendicular tumour diameters were performed every other day to estimate the tumour volume, using the following formula: 4π/3 × (width/2)^2^ × (length/2), which represents the three-dimensional volume of an ellipse. The results from the administration of GO-Y030 showed that GO-Y030 significantly suppresses (*P*<0.01) the tumour growth in SW480 (Figure 5A) and HCT-116 (Figure 6A), tumour weight in SW480 (Figure 5B) and HCT-116 (Figure 6B), and tumour mass in SW480 (Figure 5C) and HCT-116 (Figure 6C) colon cancer stem cells. The average reduction in SW480 tumour weight was 57.96% in GO-Y030-treated mice compared with the DMSO vehicle in xenograft mouse model (Figure 5B). The average reduction in HCT-116 tumour weight was 58.10% in GO-Y030-treated mice compared with the DMSO vehicle in xenograft mouse model (Figure 6B). However, the body weight of the mice treated with GO-Y030 was not reduced at the end of the treatment compared with mice treated with the DMSO vehicle (Figure 6D). These results from two independent tumour models demonstrate that GO-Y030 is potent in suppressing tumour growth from colon cancer stem cells *in vivo*.

## Discussion

Currently, the main effort to target constitutive STAT3 signalling is only focused on the bulk of cancer cells. No report has been published to target STAT3 in colon cancer-initiating cells or colon stem cells. Both CD133 and ALDH have been used to isolate colorectal cancer stem cells (O'Brien *et al*, 2007; Ricci-Vitiani *et al*, 2007; Boman and Huang, 2008). When using ALDH and CD133 together to form tumour xenografts, ALDH^+^/CD133^+^ cells showed an increased ability to generate tumour xenografts compared with ALDH^+^/CD133^–^ or ALDH^+^ alone (Huang *et al*, 2009). ALDH^+^/CD133^+^ cells tended to elicit larger tumours and elicited them more rapidly than ALDH^+^/CD133^–^ cells. Taken together, the data suggest that using both ALDH and CD133 appears to be better at enriching colorectal cancer stem cells than using ALDH or CD133. This study extends previous research by using both ALDH and CD133 together as markers for colorectal stem cells from colon cancer cell lines and examines STAT3 phosphorylation in these cancer stem cells. Our data showed that ALDH^+^/CD133^+^ cells generated more tumourspheres than ALDH^−^/CD133^−^ cells, suggesting that ALDH^+^/CD133^+^ cells possess cancer stem cell properties. Our results also indicated that colorectal cancer-initiating cells or colon stem cells, characterised by the ALDH^+^/CD133^+^ subpopulations of colorectal cancer cells, expressed higher levels of STAT3 phosphorylation than the un-separated and ALDH^−^/CD133^–^ subpopulations. These results suggest that STAT3 is a novel therapeutic target in colorectal cancer stem cells.

To explore the inhibition of STAT3 in colon cancer stem cells, we examined the inhibitory effects of a newly developed curcumin analogue, GO-Y030. Curcumin is one of the most widely characterised phytochemicals and is the active ingredient of the rhizome of the plant turmeric, which has both antioxidant and anti-inflammatory properties (Aggarwal and Shishodia, 2006). From published literature, curcumin has showed inhibitory effects in colon cancer cells (Hanif *et al*, 1997; Chauhan, 2002). Curcumin also has a chemopreventive potential in the context of colon cancer as seen in a mouse model and in human clinical trials (Kawamori *et al*, 1999; Johnson and Mukhtar, 2007). Curcumin has also been shown to inhibit STAT3 but with higher doses (Bharti *et al*, 2003; Aggarwal and Shishodia, 2006; Ohori *et al*, 2006). These results suggest that curcumin might be an ideal agent to target STAT3 in colon cancer. However, the growth suppressive activity and bioavailability of curcumin in human may still not be sufficient as an effective preventive or therapeutic agent for cancer. Therefore, more potent analogues of curcumin that can inhibit the STAT3 pathway with lower doses are needed as a more efficient form of treatments for colorectal cancer. We examined the inhibitory effects of GO-Y030 in the inhibition of STAT3 in colon cancer stem cells. GO-Y030 is one of the most potent curcumin analogues in the growth suppression of cancer cells (Ohori *et al*, 2006). Our results presented here show for the first time that GO-Y030 could efficiently inhibit STAT3 phosphorylation and cell viability, tumoursphere-forming capacity, and induce apoptosis in colorectal cancer stem cells. GO-Y030 can also downregulate putative IL-6/STAT3 downstream target genes that are involved in stem cell growth and survival such as Notch 1 (Grivennikov and Karin, 2008) as well as known STAT3 downstream target genes, such as Cyclin D1 (Bromberg *et al*, 1999), survivin (Diaz *et al*, 2006; Gritsko *et al*, 2006), Bcl-2 (Catlett-Falcone *et al*, 1999; Real *et al*, 2002), and Bcl-XL (Bromberg *et al*, 1999), that are involved in proliferation and survival. This provides possible molecular mechanisms of GO-Y030-mediated inhibition of STAT3 in colorectal cancer stem cells. Furthermore, our results show that GO-Y030 exhibits growth suppressive activity on the tumour growth of SW480 colon cancer stem cells.

These results suggested that constitutive active STAT3 in these cancer stem cells enhances proliferation and survival, as well as tumour growth in mice, whereas STAT3 blockade by GO-Y030 suppressed tumour stem cell growth *in vitro* and *in vivo*. The *in vivo* results are consistent with the *in vitro* cancer stem cell data, indicating that GO-Y030 is a potent inhibitor for the STAT3 pathway to suppress tumour growth of colon cancer stem cells in mouse models *in vivo*. In summary, this study is the first report to demonstrate that STAT3 is activated in colorectal cancer stem cells. Targeting STAT3 may be able to deplete the colorectal cancer stem cells and provide a promising approach to treat advanced colorectal cancer. Our study also demonstrated that GO-Y030 is a potent inhibiting STAT3 for cancer stem cells and is a good drug candidate to target constitutive STAT3 signalling in colorectal cancer stem cells or cancer-initiating cells.

## Figures and Tables

**Table 1:** ALDH^+^/CD133^+^ cells generated more tumourspheres than ALDH^−^/CD133^−^ cells

	**SW480**	**HCT-116**	**DLD-1**	**HT29**
*250 cells per well*				
ALDH^+^/CD133^+^	2±1^*^	2±0^*^	19±3^*^	12±2^*^
ALDH^−^/CD133^−^	0	0	0	1±1
				
*500 cells per well*				
ALDH^+^/CD133^+^	7+1^*^	8±2^*^	29±10^*^	21±3^*^
ALDH^−^/CD133^−^	0	2±0	1±1	3±2
				
*1000 cells per well*				
ALDH^+^/CD133^+^	8±2^*^	12±2^*^	42±9 ^*^	21±2^*^
ALDH^−^/CD133^−^	3±1	2±1	1±1	5±1

ALDH^+^/CD133^+^ and ALDH^−^/CD133^−^ subpopulations of colorectal cancer cells were separated by flow cytometry and cultured in stem cell medium as described in Materials and Methods. The numbers of tumoursphere generated per 250, 500, or 1000 cells were counted 2 weeks later. ^*^P<0.01.
