# Supplementary material for: Targeting colon cancer stem cells using a new curcumin analogue, GO-Y030
Source: Br J Cancer. 2011 Jun 21;105(2):212–20. doi: 10.1038/bjc.2011.200 (PMC3142799; doi:10.1038/bjc.2011.200)
Supplement: Supplementary Figures and Tables [file bjc2011200x1.ppt]

## Slide 1
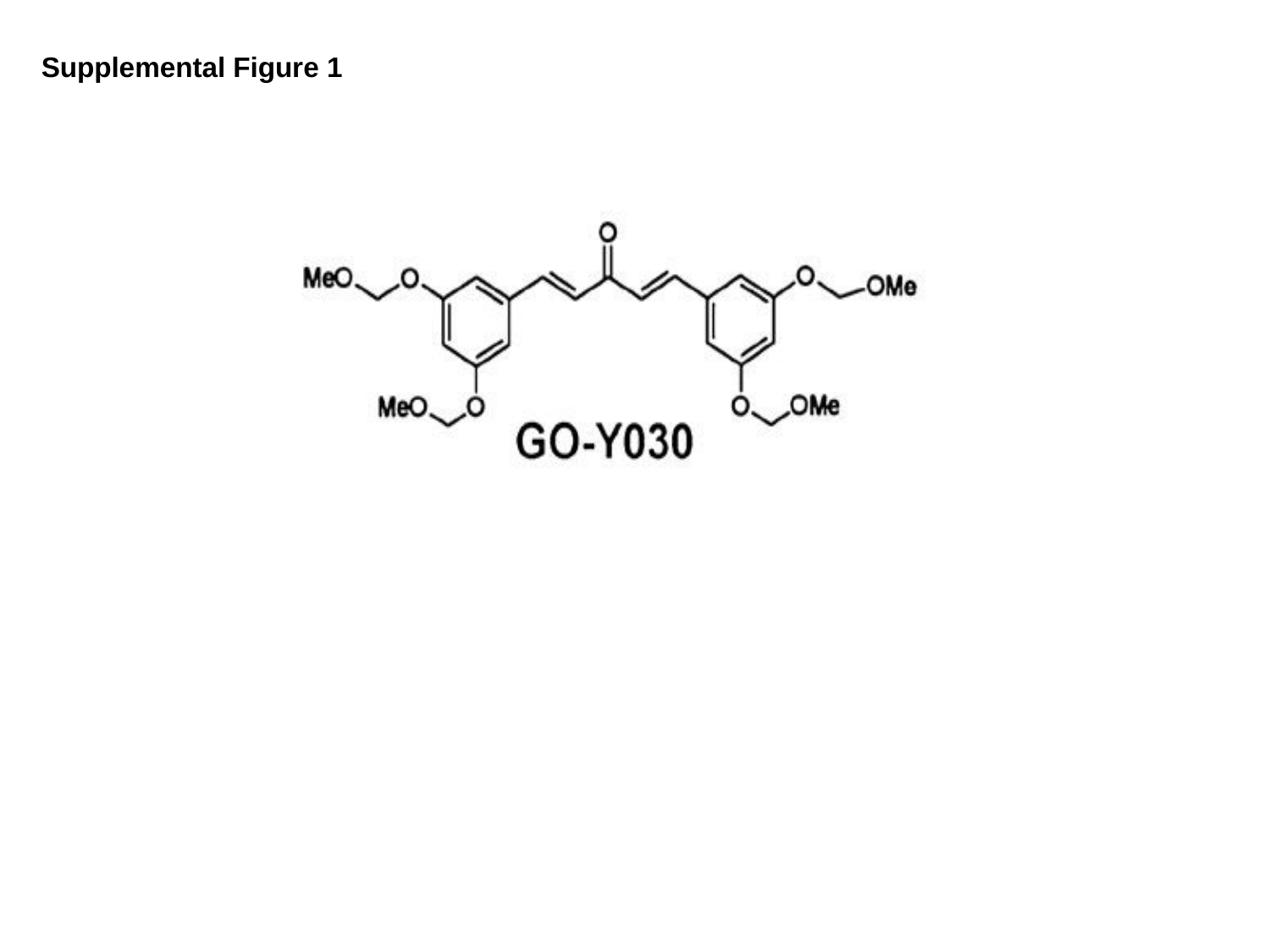

Supplemental Figure 1

## Slide 2
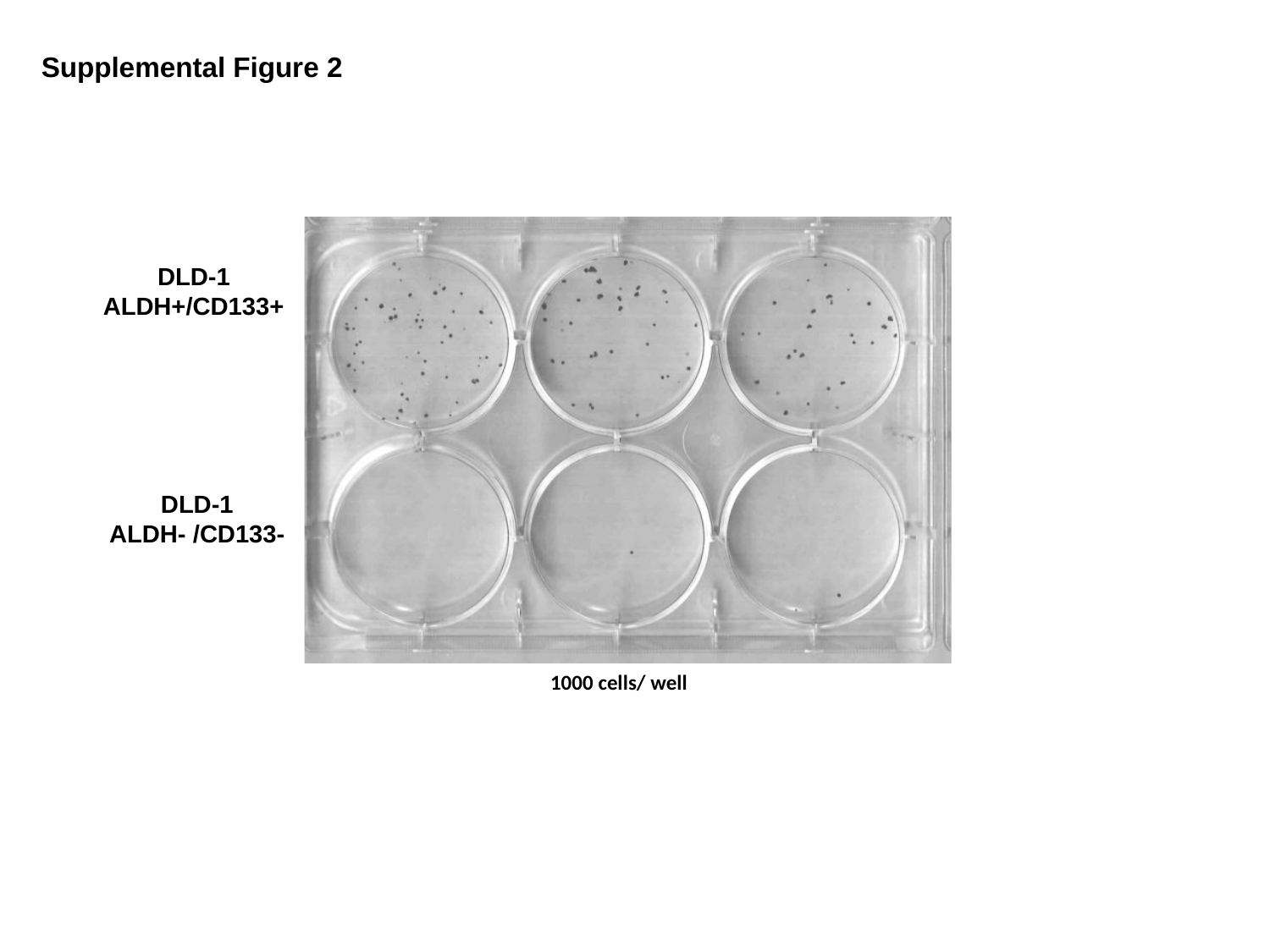

Supplemental Figure 2
DLD-1
ALDH+/CD133+
DLD-1
ALDH- /CD133-
1000 cells/ well

## Slide 3
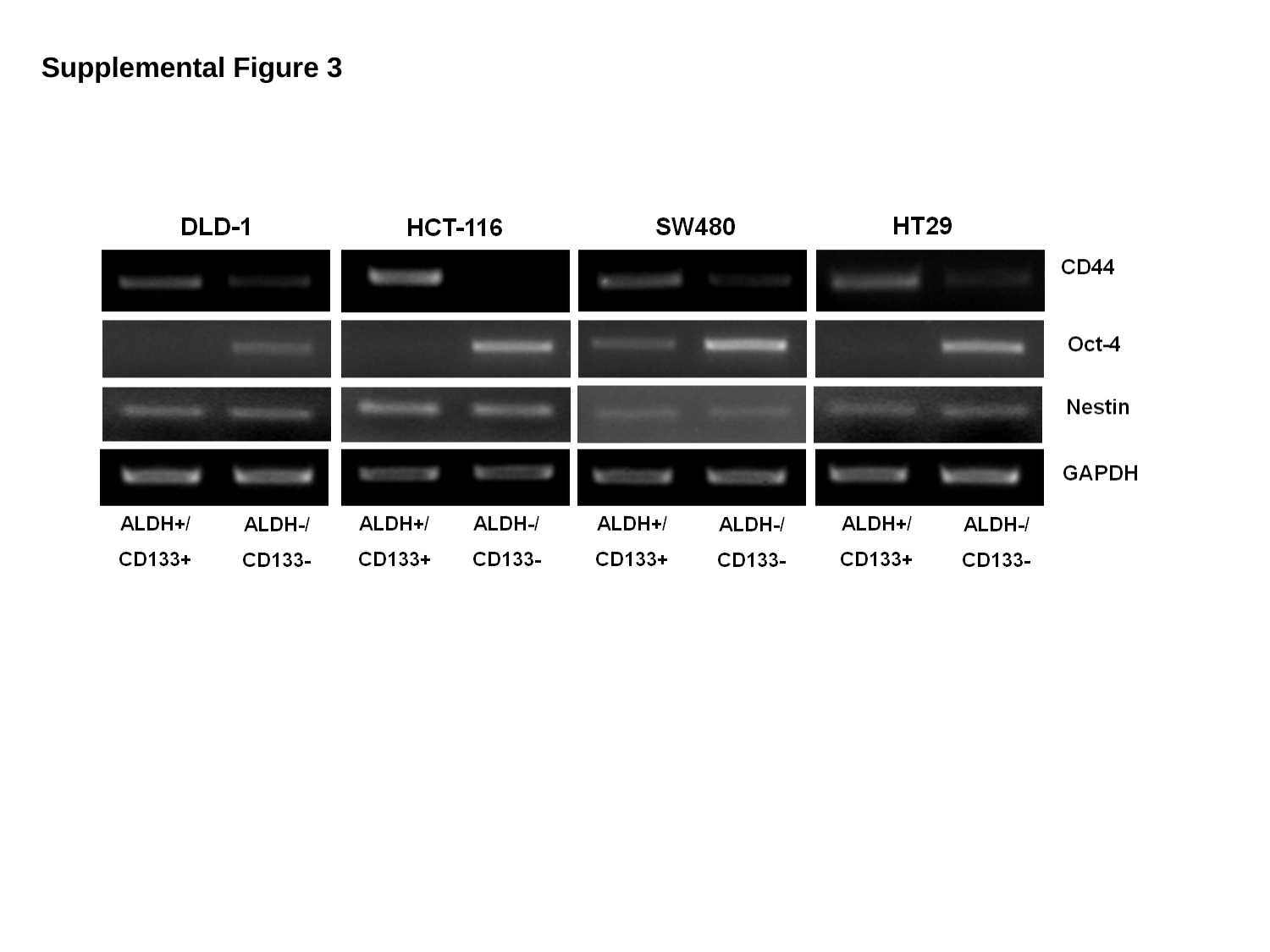

Supplemental Figure 3

## Slide 4
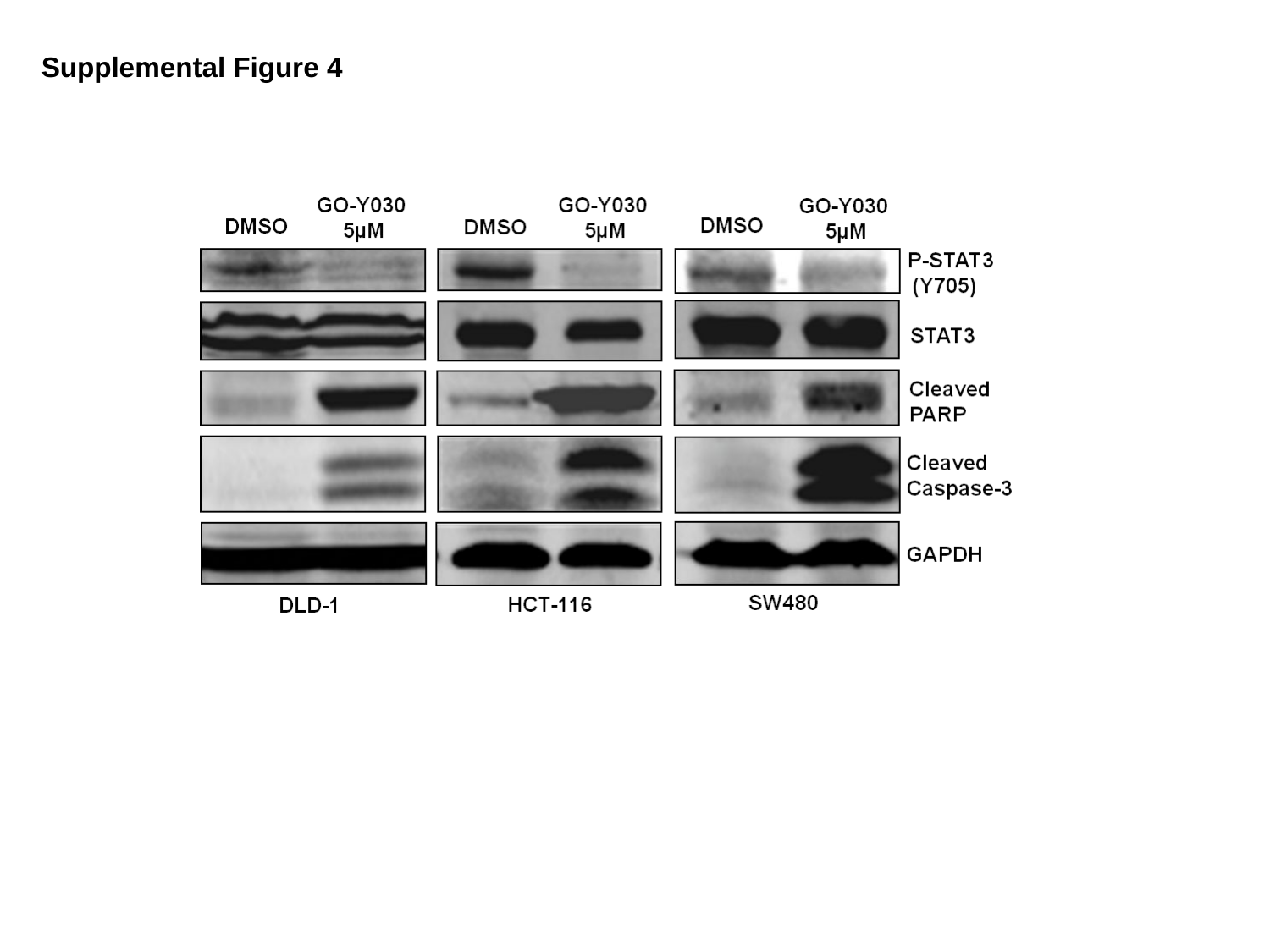

Supplemental Figure 4

## Slide 5
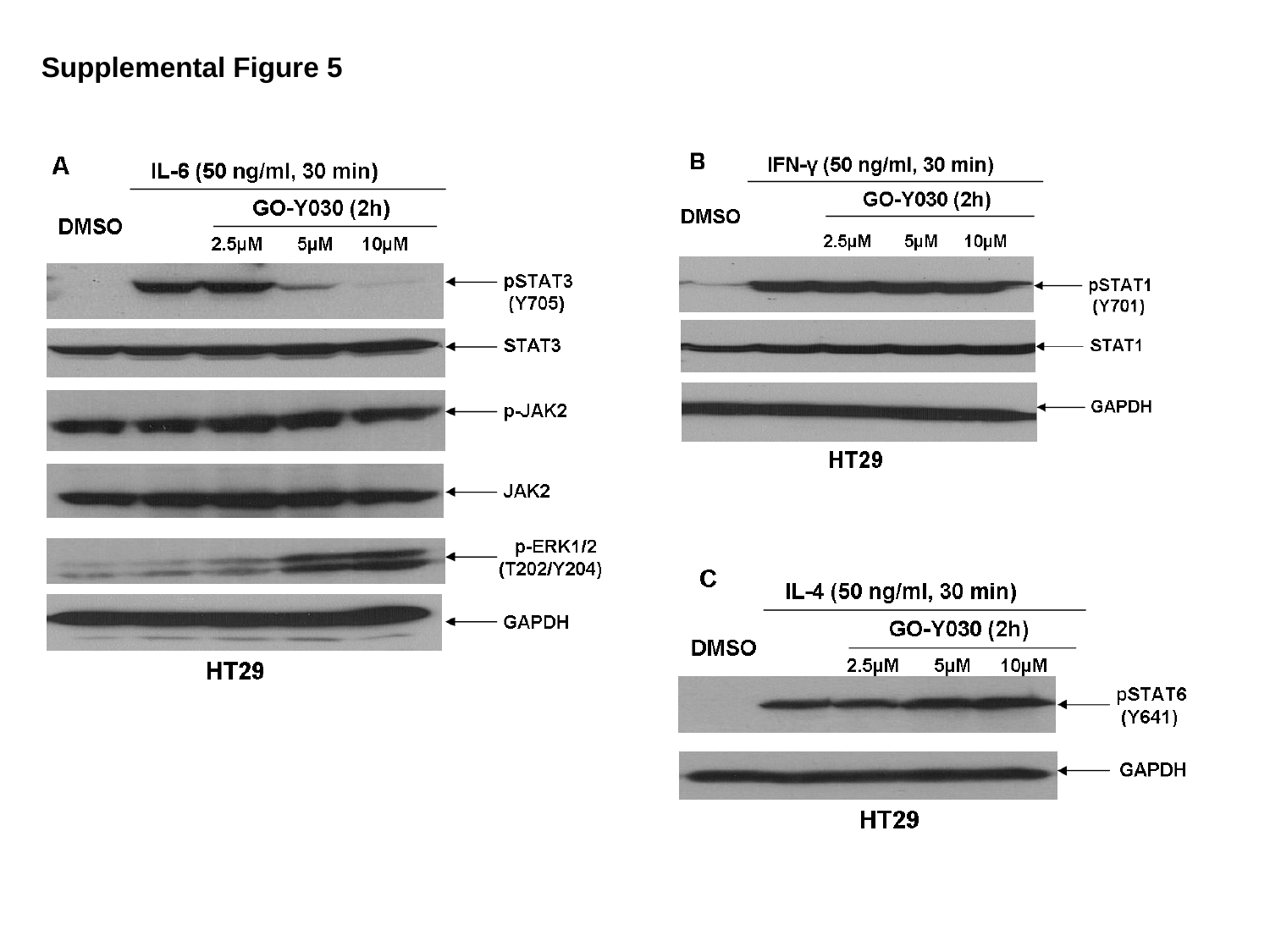

Supplemental Figure 5

## Slide 6
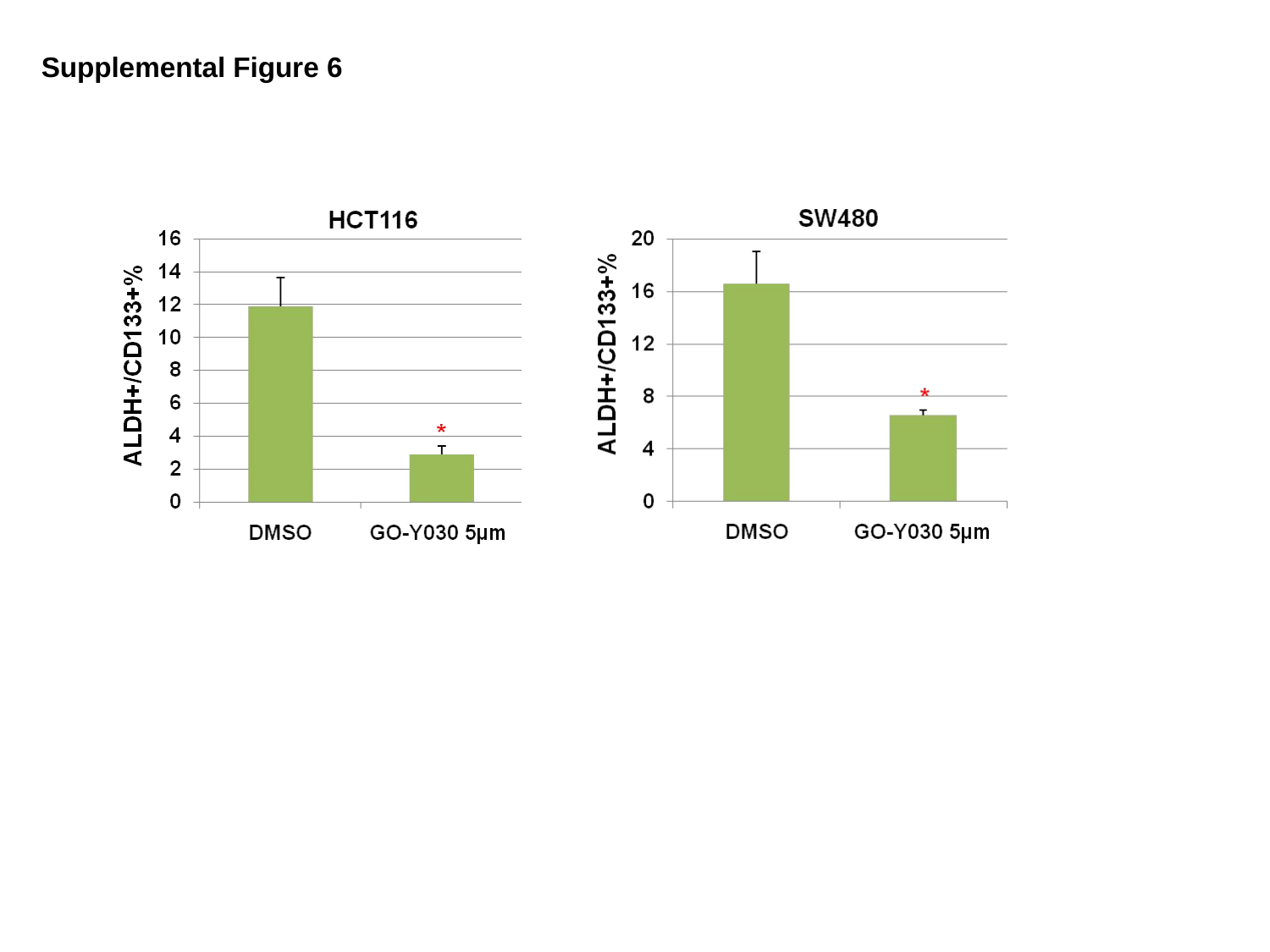

Supplemental Figure 6

## Slide 7
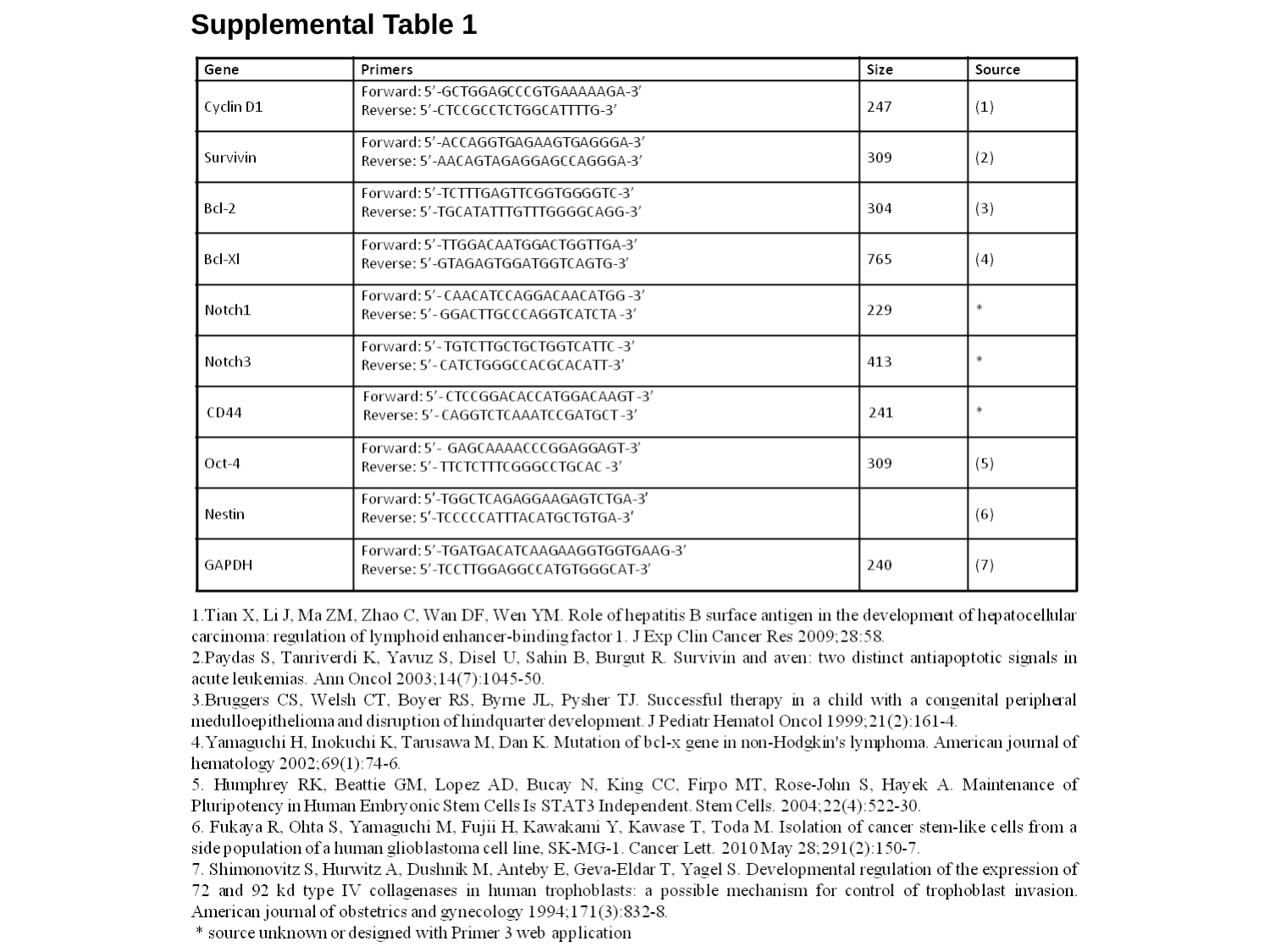

Supplemental Table 1

## Slide 8
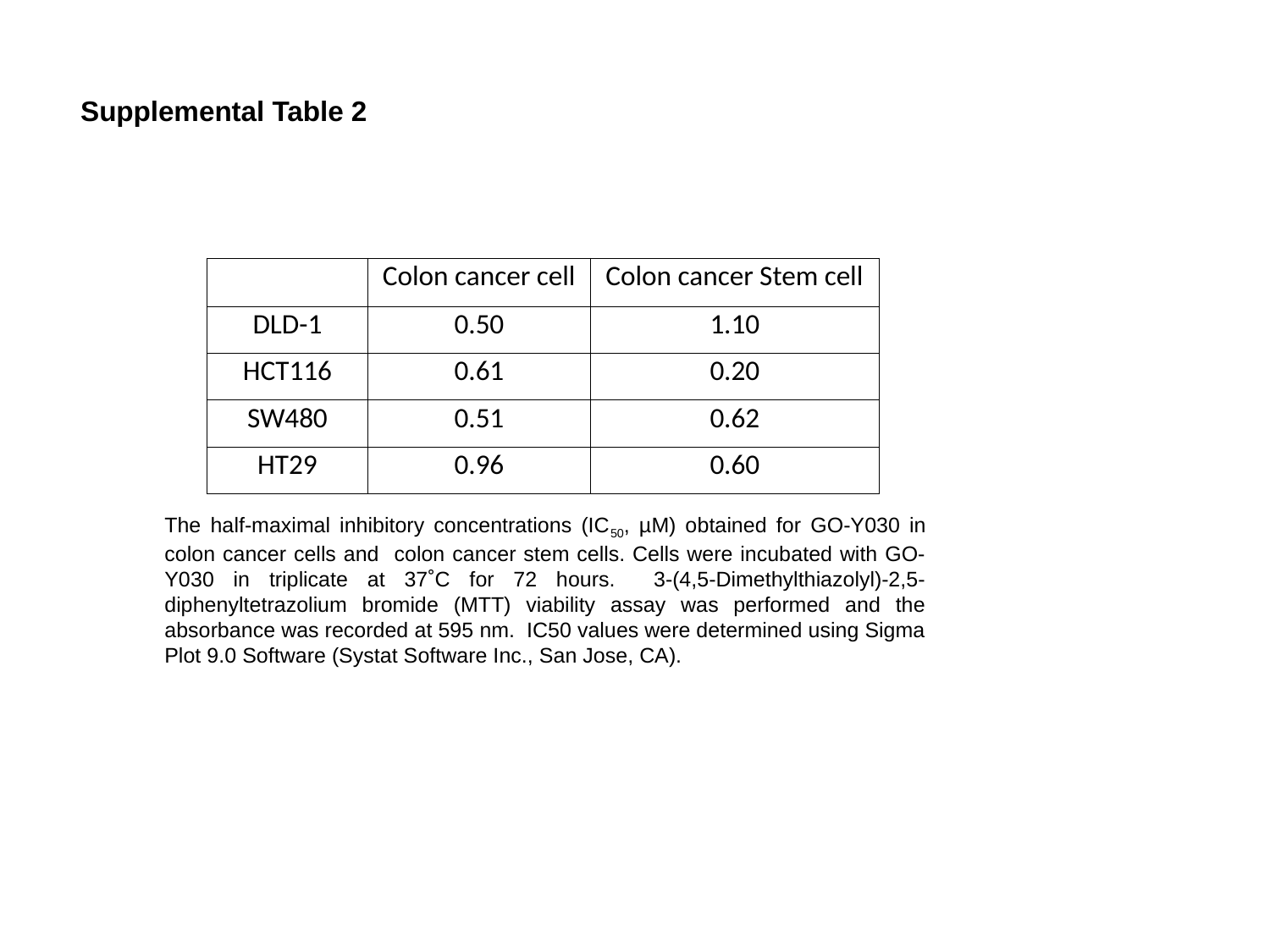

Supplemental Table 2
| | Colon cancer cell | Colon cancer Stem cell |
| --- | --- | --- |
| DLD-1 | 0.50 | 1.10 |
| HCT116 | 0.61 | 0.20 |
| SW480 | 0.51 | 0.62 |
| HT29 | 0.96 | 0.60 |
The half-maximal inhibitory concentrations (IC50, µM) obtained for GO-Y030 in colon cancer cells and colon cancer stem cells. Cells were incubated with GO-Y030 in triplicate at 37˚C for 72 hours. 3-(4,5-Dimethylthiazolyl)-2,5-diphenyltetrazolium bromide (MTT) viability assay was performed and the absorbance was recorded at 595 nm. IC50 values were determined using Sigma Plot 9.0 Software (Systat Software Inc., San Jose, CA).
