# Supplementary material for: Targeting colon cancer stem cells using a new curcumin analogue, GO-Y030
Source: Br J Cancer. 2011 Jun 21;105(2):212–20. doi: 10.1038/bjc.2011.200 (PMC3142799; doi:10.1038/bjc.2011.200)
Supplement: Supplementary Figure and Table Legends [file bjc2011200x2.doc]

**Supplemental Figure and Table legends:**

**Supplemental Figure 1.** The chemical structure of GO-Y030

**Supplemental Figure 2.** The representative tumorspheres formation from ALDH^+^/CD133^+^ or ALDH^-^/CD133^-^ subpopulations of DLD-1 colorectal cancer cells (1000 per well).

**Supplemental Figure 3.** The expression of other stem cell markers, such as CD44, Oct-4, and Nestin were tested in ALDH^+^/CD133^+^ and ALDH^-^/CD133^-^ cells were detected by RT-PCR.

**Supplemental Figure 4.** GY-Y030 inhibited STAT3 phosphorylation in un-separated human colon cancer cell lines. Cells were collected at 24 hours post-treatments. Membranes were blotted with phospho-specific STAT3 antibody (Tyrosine 705; Cell Signaling Tech.), phospho-independent STAT3 antibody (Cell Signaling Tech.), phospho-specific ERK1/2 antibody (Threonine 202/Tyrosine 204; Cell Signaling Tech.), cleaved caspase-3 antibody (Cell Signaling Tech.), cyclin D1, Bcl-2, and GAPDH antibody (Chemicon International Inc.). Colon cancer cell lines expressing constitutively active STAT3, DLD-1, HCT-116, and SW480 exhibit a decrease in the levels of expression of STAT3 phosphorylation after treatment with GO-Y030. Apoptosis is also indicated by the induction of cleaved PARP and caspase-3.

**Supplemental Figure 5. (A)** GO-Y030 (2 hours exposure at 5 or 10μM) inhibited STAT3 phosphorylation induced by IL-6 (50ng/ml) in the HT29 colon cancer cell line. GO-Y030 did not inhibit STAT3 phosphorylation and seems to enhance ERK1/2 phosphorylation. GO-Y030 also did not reduce JAK2 phosphorylation. Furthermore, GO-Y030 did not inhibit phosphorylation of STAT1 or STAT6 induced by 50ng/ml of **(B)** interferon-γ (IFN-γ) or **(C)** IL-4.

**Supplemental Figure 6.** The percentage of ALDH^+^/CD133^+^ subpopulation in HCT116 and SW480 human colon cancer cells line when treated with GO-Y030 (**P*<0.05).

**Supplemental Table 1.** The DNA sequences of primers of STAT3 downstream target genes (Cyclin D1, survivin, Bcl-2, Bcl-xL, Notch1 and Notch3) and GAPDH used for RT-PCR analysis.

**Supplemental Table 2.** The IC50s of GO-Y030 in colon cancer cells and colon cancer stem cells.

All values reflect concentrations calculated following 72 hours of treatment in an MTT viability assay.
